# Supplementary material for: Large-scale molecular endotype discovery in synovial fluid reveals osteoarthritis as a single biological continuum
Source: Nat Commun. 2026 Jun 2;17:4721. doi: 10.1038/s41467-026-71632-4 (PMC13230806; doi:10.1038/s41467-026-71632-4)
Supplement: Supplementary file 1 — Supplementary Information [file 41467_2026_71632_MOESM1_ESM.pdf]

<u>Supplementary Table 1</u>: ***Top 20 most significant proteins associated with clinical features of disease***.

	Established Radiographic OA (advanced vs. non-advanced)[1]			Disease-free controls vs all OA samples[2]		Knee Pain Subscore (WOMAC)[3]			Disease-free controls vs all OA samples[2]	
**Proteins (ordered by padj)**	**Target Name**	**Log Odds Ratio (per SD)**	**Adjusted p-value**	**Log Odds Ratio (per SD)**	**Adjusted p-value**	**Target Name**	**Beta Estimate (per SD)**	**Adjusted p-value**	**Log Odds Ratio (per SD)**	**Adjusted p-value**
**1**	TSG-6	1.04	$1.30e^{-25}$	2.15	$1.80e^{-10}$	HMCS1	6.14	$5.60e^{-12}$	0.89	$5.90e^{-06}$
**2**	sTREM-1	0.93	$1.40e^{-24}$	1.41	$4.20e^{-11}$	Activated Protein C	5.71	$4.70e^{-11}$	0.63	$1.40e^{-02}$
**3**	CI061	0.88	$1.00e^{-23}$	1.27	$4.60e^{-09}$	H31	-6.29	$5.60e^{-11}$	-0.66	$2.20e^{-04}$
**4**	RSPO2	0.99	$2.80e^{-23}$	1.25	$4.30e^{-06}$	GLCM	-5.35	$1.10e^{-10}$	-0.33	$1.50e^{-01}$
**5**	Inhibin bA chain	0.94	$6.80e^{-23}$	1.12	$6.50e^{-10}$	TTC1	5.84	$1.10e^{-10}$	0.4	$7.10e^{-02}$
**6**	PENK	0.82	$1.90e^{-22}$	0.92	$2.50e^{-07}$	CHM2B	5.61	$1.30e^{-10}$	0.18	$4.20e^{-01}$
**7**	Activin AC	0.9	$5.80e^{-22}$	1.17	$1.70e^{-10}$	FUT10	-5.34	$1.30e^{-10}$	-0.21	$3.50e^{-01}$
**8**	NELL1	0.85	$8.50e^{-22}$	1.4	$3.30e^{-08}$	6-Phosphogluconate dehydrogenase	5.6	$3.10e^{-10}$	0.64	$7.60e^{-03}$
**9**	Activin A	0.89	$4.50e^{-21}$	0.88	$1.80e^{-09}$	CCD50	5.34	$3.40e^{-10}$	0.08	$7.60e^{-01}$
**10**	Tenascin	0.81	$4.00e^{-20}$	1.5	$3.70e^{-10}$	Aminoacylase-1	5.99	$1.20e^{-09}$	0.02	$9.30e^{-01}$
**11**	ELA1	0.75	$5.10e^{-20}$	1.32	$1.80e^{-10}$	KLC1	5.14	$1.60e^{-09}$	0.66	$1.70e^{-02}$
**12**	Tenascin	0.78	$5.40e^{-20}$	1.29	$3.30e^{-10}$	ERP29	5.81	$4.00e^{-09}$	0.61	$3.40e^{-03}$
**13**	Tenascin	0.72	$7.60e^{-20}$	1.03	$6.70e^{-11}$	EIF1B	5.28	$4.10e^{-09}$	0.38	$1.10e^{-01}$
**14**	Spondin-1	0.78	$2.50e^{-19}$	1.26	$9.10e^{-09}$	NOE2	6.05	$1.40e^{-08}$	0.29	$7.30e^{-02}$
**15**	F176C:CD	0.72	$1.20e^{-18}$	1.19	$2.80e^{-10}$	CI061	5.31	$1.40e^{-08}$	1.27	$4.60e^{-09}$
**16**	Spondin-1	0.77	$1.60e^{-18}$	1.43	$1.80e^{-08}$	ARLY	5.84	$1.50e^{-08}$	0.51	$3.00e^{-03}$
**17**	ON	0.77	$2.10e^{-18}$	0.93	$5.10e^{-06}$	LDHA	5.36	$1.80e^{-08}$	0.44	$1.90e^{-03}$
**18**	PLOD3	0.77	$3.00e^{-18}$	1.05	$2.60e^{-06}$	IDH	5.81	$5.90e^{-08}$	0.29	$1.00e^{-01}$
**19**	CATO	0.75	$3.70e^{-18}$	0.79	$8.20e^{-06}$	NDRG2	-4.74	$5.90e^{-08}$	-0.12	$5.80e^{-01}$
**20**	GFPT2	0.85	$8.90e^{-18}$	0.82	$2.50e^{-03}$	LKHA4	-4.72	$6.90e^{-08}$	-0.7	$5.80e^{-05}$

Top 20 most significant proteins (ordered by adjusted p-value in each of the respective analyses): i) advanced radiographic disease status and ii) WOMAC knee pain subscore with accompanying log odds ratios between categories per standard deviation change and adjusted p-values (Benjamini-Hochberg adjusted, padj) in the disease status (OA vs DF-controls) analysis for the corresponding protein. All values reported here have been rounded to 2 decimal places.

[1] Model: logistic regression using Combined dataset (N = 1,322 OA samples) (ComBat corrected for spin-status, non-IPS regressed), adjusted for participant age and biological sex.
Outcome: advanced (KL grades: 3-4, n = 1016) vs. non-advanced (KL grades: 0-2, n = 306).

[2] Model: linear regression using Combined dataset (N = 805 OA samples) (ComBat corrected for spin-status, non-IPS regressed), adjusted for participant age and biological sex.
Outcome: WOMAC knee pain subscore, 0-100 scores (100 = worst knee pain).

[3] Model: logistic regression using Combined dataset (N = 1397 samples (1361 OA vs. 36 DF-control samples)) (ComBat corrected for spin-status, non-IPS regressed), adjusted for participant age and biological sex.
Outcome: disease status (OA vs DF-controls).

Abbreviations: intracellular protein score (IPS); adjusted p-value (padj); standard deviation (SD); disease-free (DF); osteoarthritis (OA); Western Ontario and McMaster Universities Osteoarthritis Index (WOMAC); Kellgren Lawrence (KL).

STEpUP OA DATASETS

			DISCOVERY	REPLICATION	COMBINED	
			**Baseline OA Samples**			**Baseline Disease-Free Control Samples**
			N = 719 (***before*** *filtering*)[1]	N = 664 (***before*** *filtering*)[1]	N = 1,383 (***before*** *filtering*)[1]	N = 37 (***before*** *filtering*)[1]
			N = 708 (***after*** *filtering*)[2]	N = 653 (***after*** *filtering*)[2]	N = 1,361 (***after*** *filtering*)[2]	N = 36 (***after*** *filtering*)[2]
**Feature**	**Feature Description**	**Number of samples with available data (Discovery, Replication and Combined datasets)**	**Mean (SD) or N (%)**			**Number of samples with available data & Mean (SD) or N (%)**
**Age**	Participant age at the time of sampling (year)	707, 653, 1360	64.4 (11.5)	65.5 (10.3)	64.9 (11)	N = 35, 63.8 (9.7)
**Sex**	Biological sex	708, 653, 1361	Female = 366 (52%), Male = 342 (48%)	Female = 344 (53%), Male = 309 (47%)	Female = 710 (52%), Male = 651 (48%)	N = 35, Female = 11 (31%) Male = 24 (69%)
**BMI**	Participant body mass index at the time of sampling	694, 542, 1236	30.6 (5.9)	30.6 (5.6)	30.6 (5.8)	N = 30, 27.7 (4.3)
**Smoking History**	Current or past smoker at the time of the baseline sampling	623, 477, 1100	Never smoked: N = 332 (53%) Ever smoked: N = 291 (47%)	Never smoked: N = 267 (56%) Ever smoked: N = 210 (44%)	Never smoked: N = 599 (55%) Ever smoked: N = 501 (45%)	NA
**WOMAC Pain Score**	Scale of 0-100, where 100 is the worst possible knee pain	420, 385, 805	48.7 (20.7)	35.9 (23)	42.6 (22.7)	N = 30, 5.8 (14.9)
**Advanced Radiographic Status**	Binary indicator for the presence of advanced radiographic knee OA (KL grades 3-4)	705, 617, 1322	Non-advanced: N = 131 (19%) Advanced: N = 574 (81%)	Non-advanced: N = 175 (28%) Advanced: N = 442 (72%)	Non-advanced: N = 306 (23%) Advanced: N = 1016 (77%)	N = 29, Non-advanced: N = 29 (100%)

**Ordinal KL Grade**	Kellgren Lawrence grade (0-4) (worst affected compartment)	187, 579, 766	0: 19 (10%)<br>1: 35 (19%)<br>2: 65 (35%)<br>3: 43 (23%)<br>4: 25 (13%)	0: 15 (3%)<br>1: 59 (10%)<br>2: 101 (18%)<br>3: 234 (40%)<br>4: 170 (29%)	0: 34 (4%)<br>1: 94 (12%)<br>2: 166 (22%)<br>3: 277 (36%)<br>4: 195 (26%)	N = 28,<br>0: 28 (100%)
**Spin Status**	Binary indicator for whether SF sample had been centrifuged prior to supernatant storage	708, 653, 1361	Unspun: N = 1 (0.1%)<br>Spun: N = 707 (99.9%)	Unspun: N = 226 (35%)<br>Spun: N = 427 (65%)	Unspun: N = 227 (17%)<br>Spun: N = 1134 (83%)	N = 36,<br>Unspun: N = 30 (83%)<br>Spun: N = 6 (17%)

[1] Total number of individual baseline (first available visit) OA synovial fluid samples (or disease-free control samples) before additional QC sample filtering.
[2] Total number of individual baseline OA synovial fluid samples (or disease-free control samples) after additional QC sample filtering (N = 15 samples were removed from Discovery & N = 11 from Replication datasets) – samples of poor quality were removed.

<u>Supplementary Table 2</u>: ***Characteristics of Discovery, Replication and Combined datasets and participant samples.*** Demographic and clinical data are summarized. Abbreviations: quality control (QC); Kellgren Lawrence (KL); Western Ontario and McMaster Universities Osteoarthritis Index (WOMAC); standard deviation (SD); body mass index (BMI); centrifuged ('spun'); unspun ('non-centrifuged'); 'advanced' (KL grades: 3-4); 'non-advanced' (KL grades: 0-2).

Supplementary Table 3: ***Characteristics of STEpUP OA analyses.***

Figure	Analysis / Visualisation	Disease Group	Number of samples included (n)	Dataset	Number of SOMAmers (n)	Stratification	Model adjustment(s)
**Figure 1**							
**Figure 1A, 1B**	Cluster analysis: (f(K) statistic) & Visualisation on UMAP	OA	Baseline OA samples (spun) Discovery, N = 707 Replication, N = 427 Combined, N = 1134	non-IPS, IPS regressed	non-IPS, N = 6558 IPS regressed, N = 6290	Discovery, Replication, Combined datasets	N/A
**Figure 1C**	Cluster analysis: (f(K) statistic)	OA	Baseline OA samples (spun) Female, N = 596 Male, N = 538	non-IPS, IPS regressed	non-IPS, N = 6558 IPS regressed, N = 6290	Combined datasets by biological sex	N/A
**Figure 1D**	Cluster analysis: (f(K) statistic)	OA	Baseline OA samples (spun) Non-advanced radiographic disease, N = 122 Advanced radiographic disease, N = 832	non-IPS, IPS regressed	non-IPS, N = 6558 IPS regressed, N = 6290	Combined datasets by advanced / non-advanced radiographic disease status	N/A
**Figure 1E**	Cluster analysis: (f(K) statistic)	OA	Baseline OA samples (spun) No blood staining, N = 394 With blood staining, N = 121	non-IPS, IPS regressed	non-IPS, N = 6558 IPS regressed, N = 6290	Combined datasets by visual blood staining score ('no blood staining' vs 'with blood staining')	N/A
**Figure 2**							
**Figure 2A**	Volcano plot: associations with advanced x-ray status	OA	Baseline OA samples (spun & unspun) N = 1,322	Combined, ComBat spin-status corrected, non-IPS regressed	5471	N/A	Age, biological sex

**Figure 2B**	Boxplots: associations with ordinal KL grade for top associating proteins	OA	Baseline OA samples (spun & unspun) N = 766	Combined, ComBat spin-status corrected, non-IPS regressed	N/A	N/A	Age, biological sex
**Figure 2C**	Bubble plot: fgsea of associations with advanced x-ray status	OA	Baseline OA samples (spun & unspun) Discovery, N = 705 Replication, N = 617 Combined, N = 1322	ComBat spin-status corrected, non-IPS regressed	5471	Discovery, Replication, Combined	Age, biological sex
**Figure 2D, Figure 2E, Figure 2F**	STRING plots: Epithelial to Mesenchymal Pathway, Complement Pathway, Angiogenesis Pathway	OA	Baseline OA samples (spun & unspun) N = 1,322	Combined, ComBat spin-status corrected, non-IPS regressed	5471 (top 1000 proteins, ordered by logOR, passed to STRING)	N/A	Age, biological sex
**Figure 2G**	Pearson correlation: comparing log odds ratios from models using Discovery or Replication datasets for the outcome: advanced x-ray status	OA	Baseline OA samples (spun & unspun) Discovery, N = 705 Replication, N = 617	ComBat spin-status corrected, non-IPS regressed	5471	Discovery, Replication	Age, biological sex
**Figure 3**							
**Figure 3A, 3B**	Volcano plot: associations with advanced x-ray status (obese participants only or non-obese participants only)	OA	Baseline OA samples (spun & unspun) Obese participants, N = 587 or Non-obese participants, N = 649	Combined, ComBat spin-status corrected, non-IPS regressed	5471	N/A	Age, biological sex
**Figure 3C**	Pearson correlation: comparing log odds ratios from models using Combined (obese only) and Combined (non-obese only) datasets for the outcome: advanced x-ray status	OA	Baseline OA samples (spun & unspun) Obese participants, N = 587 Non-obese participants, N = 649	Combined, ComBat spin-status corrected, non-IPS regressed	5471	Obese, non-obese	Age, biological sex

**Figure 3D**	Bubble plot: fgsea of associations with advanced x-ray status by obesity status	OA	Baseline OA samples (spun & unspun) Obese participants, N = 587 Non-obese participants, N = 649	Combined, ComBat spin-status corrected, non-IPS regressed	5471	Obese, Non-obese	Age, biological sex
**Figure 4**							
**Figure 4A, 4B**	Volcano plot: associations with advanced x-ray status (males only or females only)	OA	Baseline OA samples (spun & unspun) Male participants, N = 623 or Female participants, N = 699	Combined, ComBat spin-status corrected, non-IPS regressed	5471	N/A	Age
**Figure 4C**	Pearson correlation: comparing log odds ratios from models using Combined (males only) and Combined (females only) datasets for the outcome: advanced x-ray status	OA	Baseline OA samples (spun & unspun) Males, N = 623 Females, N = 699	Combined, ComBat spin-status corrected, non-IPS regressed	5471	Males, females	Age
**Figure 4D**	Bubble plot: fgsea of associations with advanced x-ray status by biological sex	OA	Baseline OA samples (spun & unspun) Males, N = 623 Females, N = 699	Combined, ComBat spin-status corrected, non-IPS regressed	5471	Males, females	Age vs. age & log(haemoglobin A)
**Figure 5**							
**Figure 5A**	Volcano plot: associations with WOMAC knee pain	OA	Baseline OA samples (spun & unspun) N = 805	Combined, ComBat spin-status corrected, non-IPS regressed	5471	N/A	Age, biological sex
**Figure 5B**	Scatter plot: Comparing beta estimates from models using Discovery	OA	Baseline OA samples (spun & unspun) Discovery, N = 420	ComBat spin-status corrected, non-IPS regressed	5471	Discovery, Replication	Age, biological sex

	and Replication datasets for the outcome: WOMAC knee pain		Replication, N = 385				
**Figure 5C**	Scatter plots: log(NOE2) expression against WOMAC pain subscores // log(NAR3) expression against WOMAC pain subscores	OA	Baseline OA samples (spun & unspun) N = 805	Combined, ComBat spin-status corrected, non-IPS regressed	Noelin-2 (NOE2) or Ecto-ADP-ribosyltransferase 3 (NAR3)	N/A	Age, biological sex
**Figure 5D**	Bubble plot: fgsea of associations with WOMAC knee pain	OA	Baseline OA samples (spun & unspun) Discovery, N = 420 Replication, N = 385 Combined, N = 805	ComBat spin-status corrected, non-IPS regressed	5471	Discovery, Replication, Combined	Age, biological sex
**Supplementary Figure 1**							
**Supplementary Figure 1A**	Volcano plot: associations with advanced x-ray status with paired cartilage RNA-seq genes taken from Soul *et al*. 2018 overlaid	OA	STEpUP OA Baseline OA samples (spun & unspun) N = 1,322	Combined, ComBat spin-status corrected, non-IPS regressed	Association testing in 5471 SOMAmers, 4832 mapped to cartilage RNA-seq	NA	Age, biological sex
	Bubble plot: fgsea of cartilage RNA-seq dataset taken from Soul *et al*. 2018 overlaid	NA	NA	NA	48428 genes (with log_2 fold change and adjusted p-values available)	NA	NA
**Supplementary Figure 1B**	Volcano plot: associations with advanced x-ray status with paired synovium RNA-seq genes taken from Guo *et al*. 2017 overlaid	OA	STEpUP OA Baseline OA samples (spun & unspun) N = 1,322	Combined, ComBat spin-status corrected, non-IPS regressed	Association testing in 5471 SOMAmers, 4364 mapped to synovium RNA-seq	NA	Age, biological sex
	Bubble plot: fgsea of synovium RNA-seq	NA	NA	NA	25022 genes (with log_2 fold	NA	NA

	dataset taken from Guo *et al*. 2017 overlaid				change and adjusted p-values available)		
**Supplementary Figure 2**							
**Supplementary Figure 2A**	Scatter plot: comparing log odds ratios from models using Combined dataset with and without adjustment for cohort for the outcome: advanced x-ray status	OA	Baseline OA samples (spun & unspun) N = 1,322	Combined, ComBat spin-status corrected, non-IPS regressed	5471	With/without adjustment for cohort	Age, biological sex vs. Age, biological sex and cohort
**Supplementary Figure 2B**	Pie chart: cohort composition for advanced x-ray status by Discovery & Replication datasets	OA	Baseline OA samples (spun & unspun) Discovery, N = 705 Replication, N = 617	N/A	N/A	By cohort; within Discovery and Replication datasets, respectively	N/A
**Supplementary Figure 2C**	Bubble plot: fgsea of associations with advanced x-ray status (adjusted for cohort)	OA	Baseline OA samples (spun & unspun) Discovery, N = 705 Replication, N = 617 Combined, N = 1322	ComBat spin-status corrected, non-IPS regressed	5471	Discovery, Replication, Combined	Age, biological sex, cohort
**Supplementary Figure 3**							
**Supplementary Figure 3A**	Volcano plot: associations with advanced x-ray status	OA	Baseline OA samples (spun & unspun) N = 1,322	Combined, ComBat spin-status corrected, IPS regressed	5278	N/A	Age, biological sex
**Supplementary Figure 3B**	Pearson correlation: comparing log odds ratios from models using Combined dataset with and without adjustment for IPS for the outcome: advanced x-ray status	OA	Baseline OA samples (spun & unspun) N = 1,322	Combined, ComBat spin-status corrected	5210 (proteins common in both non-IPS and IPS regressed analyses)	With/without adjustment for IPS	Age, biological sex
**Supplementary Figure 3C**	Bubble plot: fgsea of associations with advanced x-ray status	OA	Baseline OA samples (spun & unspun) Discovery, N = 705	ComBat spin-status corrected, IPS regressed	5278	Discovery, Replication, Combined	Age, biological sex

			Replication, N = 617<br>Combined, N = 1322				
**Supplementary Figure 4**							
**Supplementary Figure 4A**	Volcano plot: associations with disease status (comparing proteomes of OA vs disease-free controls)	OA & Disease-free controls	Baseline samples (spun & unspun)<br>OA: N = 1,361,<br>Disease-free controls: N = 36	Combined, ComBat spin-status corrected, non-IPS regressed	5471	N/A	Age, biological sex
**Supplementary Figure 4B**	Bubble plot: fgsea of associations with disease status (comparing proteomes of OA vs disease-free controls)	OA & Disease-free controls	Baseline samples (spun & unspun)<br>Discovery: OA, N = 708; N = 36 DF-controls<br>Replication: OA, N = 653; N = 36 DF-controls<br>Combined: OA, N = 1361; N = 36 DF-controls	ComBat spin-status corrected, non-IPS regressed	5471	Discovery, Replication, Combined	Age, biological sex
**Supplementary Figure 4C**	Scatter plot: comparing log odds ratios from analyses of advanced x-ray status and disease status (OA vs DF-controls) outcomes	OA & Disease-free controls	Baseline OA samples (spun & unspun)<br>N = 1,322 vs.<br>Baseline samples (spun & unspun)<br>OA: N = 1,361,<br>Disease-free controls: N = 36	Combined, ComBat spin-status corrected, non-IPS regressed	5471	N/A	Age, biological sex
**Supplementary Figure 5**							
**Supplementary Figure 5A**	Volcano plot: associations with BMI (continuous)	OA	Baseline OA samples (spun & unspun)<br>N = 1236	Combined, ComBat spin-status corrected, non-IPS regressed	5471	N/A	Biological sex, advanced x-ray status
**Supplementary Figure 5B**	Pearson correlation: participant BMI against log(leptin protein expression)	OA	Baseline OA samples (spun & unspun)<br>N = 1236	Combined, ComBat spin-status corrected, non-IPS regressed	Leptin	N/A	NA
**Supplementary Figure 5C**	STRING plot: associations with BMI	OA	Baseline OA samples (spun & unspun)<br>N = 1236	Combined, ComBat spin-status corrected, non-IPS regressed	5471<br>(top 50 proteins, ordered by beta	N/A	Biological sex, advanced x-ray status

					estimate, passed to STRING)		
**Supplementary Figure 5D**	Scatter plot: comparing beta estimates from models using Combined dataset with and without adjustment for cohort for the outcome: BMI (continuous)	OA	Baseline OA samples (spun & unspun) N = 1236	Combined, ComBat spin-status corrected, non-IPS regressed	5471	With/without adjustment for cohort	Age, biological sex vs. Age, biological sex and cohort
**Supplementary Figure 5E**	Density plot: distribution in BMI by cohort across Discovery and Replication datasets	OA	Baseline OA samples (spun & unspun) N = 1236	NA	N/A	By cohort; within Discovery and Replication datasets, respectively	N/A
**Supplementary Figure 6**							
**Supplementary Figure 6A**	Boxplot: log(CRP protein expression) by quartiles	OA	Baseline OA samples (spun & unspun) N = 1361	Combined, ComBat spin-status corrected, non-IPS regressed	C-reactive protein (CRP)	N/A	N/A
**Supplementary Figure 6B**	Boxplot: association between log(CRP) and ordinal KL grade	OA	Baseline OA samples (spun & unspun) N = 766	Combined, ComBat spin-status corrected, non-IPS regressed	C-reactive protein (CRP)	N/A	Age, biological sex
**Supplementary Figure 6C**	Scatter plot: log(CRP) expression against WOMAC pain subscores	OA	Baseline OA samples (spun & unspun) N = 805	Combined, ComBat spin-status corrected, non-IPS regressed	C-reactive protein (CRP)	N/A	Age, biological sex, advanced x-ray status
**Supplementary Figure 6D**	Volcano plot: associations with log(CRP)	OA	Baseline OA samples (spun & unspun) N = 1361	Combined, ComBat spin-status corrected, non-IPS regressed	5470	N/A	Age, biological sex, advanced x-ray status
**Supplementary Figure 6E**	Bubble plot: fgsea of associations with log(CRP)	OA	Baseline OA samples (spun & unspun) Combined, N = 1361	ComBat spin-status corrected, non-IPS regressed	5470	Combined	Age, biological sex, advanced x-ray vs.

							Age, biological sex, advanced x-ray, BMI
**Supplementary Figure 7**							
**Supplementary Figure 7A**	Volcano plot: associations with WOMAC knee pain (adjusting for cohort)	OA	Baseline OA samples (spun & unspun) N = 805	Combined, ComBat spin-status corrected, non-IPS regressed	5471	N/A	Age, biological sex, cohort
**Supplementary Figure 7B**	Density plot: distribution in WOMAC knee pain subscores by cohort across Discovery and Replication datasets	OA	Baseline OA samples (spun & unspun) Discovery, N = 420 Replication, N = 385	NA	N/A	By cohort; within Discovery and Replication datasets, respectively	N/A
**Supplementary Figure 7C**	Volcano plot: associations with WOMAC knee pain (adjusting for advanced x-ray status)	OA	Baseline OA samples (spun & unspun) N = 773	Combined, ComBat spin-status corrected, non-IPS regressed	5471	N/A	Age, biological sex, advanced x-ray status
**Supplementary Figure 7D**	Boxplots: log(NOE2) expression against ordinal KL grade // log(NAR3) expression against ordinal KL grade	OA	Baseline OA samples (spun & unspun) N = 766	Combined, ComBat spin-status corrected, non-IPS regressed	Noelin-2 (NOE2) or Ecto-ADP-ribosyltransferase 3 (NAR3)	N/A	Age, biological sex
**Supplementary Figure 7E**	Scatter plot: comparing beta estimates from models using Combined dataset with and without adjustment for IPS for the outcome: WOMAC knee pain	OA	Baseline OA samples (spun & unspun) N = 805	Combined, ComBat spin-status corrected	5210 (proteins common in both non-IPS and IPS regressed analyses)	With/without adjustment for IPS	Age, biological sex

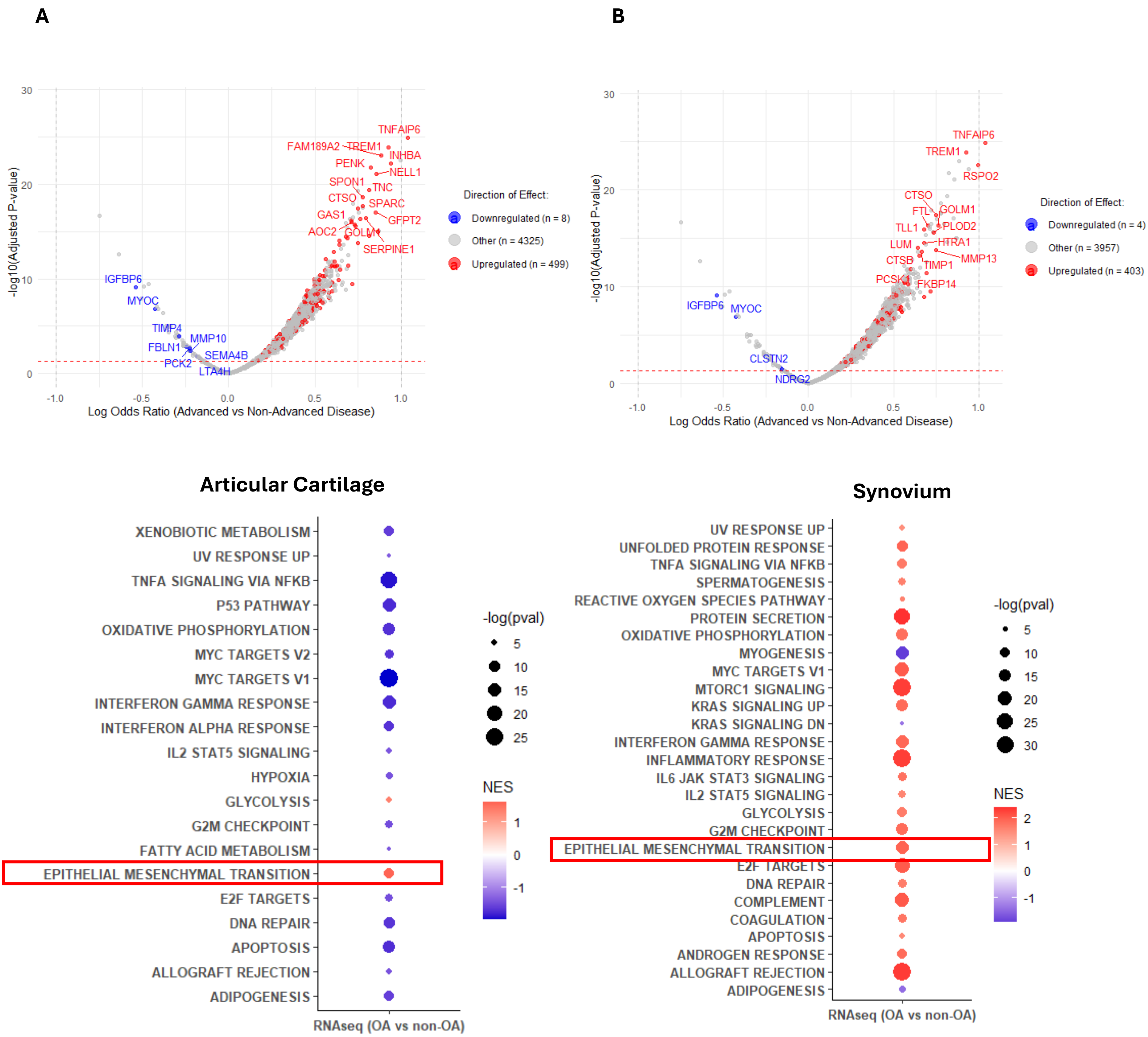

<u>Supplementary Figure 1</u>: ***Overlap between RNA-seq gene expression in diseased (OA) articular cartilage or diseased (OA) synovium and protein abundance measured in synovial fluid in advanced radiographic knee OA status from the STEpUP OA dataset*.**

Protein abundance was measured in 1,322 samples (1,096 spun, 226 unspun), with associations between protein abundance and advanced radiographic disease tested using logistic regression modelling; corrected for spin-status by ComBat, and then adjusted for age and biological sex. The dataset included 1,016 advanced OA cases and 306 non-advanced cases. **(A) Cartilage:** the volcano plot shows proteins associated with advanced radiographic OA in the Combined dataset, with log odds ratios (logOR) between categories per standard deviation change in SF protein expression plotted against adjusted p-values (Benjamini-Hochberg adjusted, padj). Proteins in red are positively

associated with advanced radiographic disease in synovial fluid and upregulated in diseased (OA) cartilage (at padj ≤ 0.05), while those in blue are negatively associated. The top 15 positively associated and 8 negatively associated proteins (by padj) (in both SF and diseased cartilage) are labelled. The bubble plot shows significantly enriched pathways (padj < 0.05) in OA articular cartilage, compared to the non-OA group, using the Hallmark Gene set (N = 48,428 genes). **(B) Synovium:** the volcano plot shows proteins associated with advanced radiographic OA in synovial fluid, with proteins in red positively associated with advanced radiographic disease and upregulated in diseased (OA) synovium (at padj ≤ 0.05), while blue proteins are negatively associated. The top 15 positively and 4 negatively associated proteins (by padj) (in both SF and diseased synovium) are labelled. The bubble plot shows enriched pathways (by padj) in diseased synovium, compared to the non-OA group, using the Hallmark Gene set (N = 25,022 genes). Abbreviations: normalised enrichment score (NES); RNA sequencing (RNA-seq); osteoarthritis (OA); synovial fluid (SF); adjusted p-value (padj).

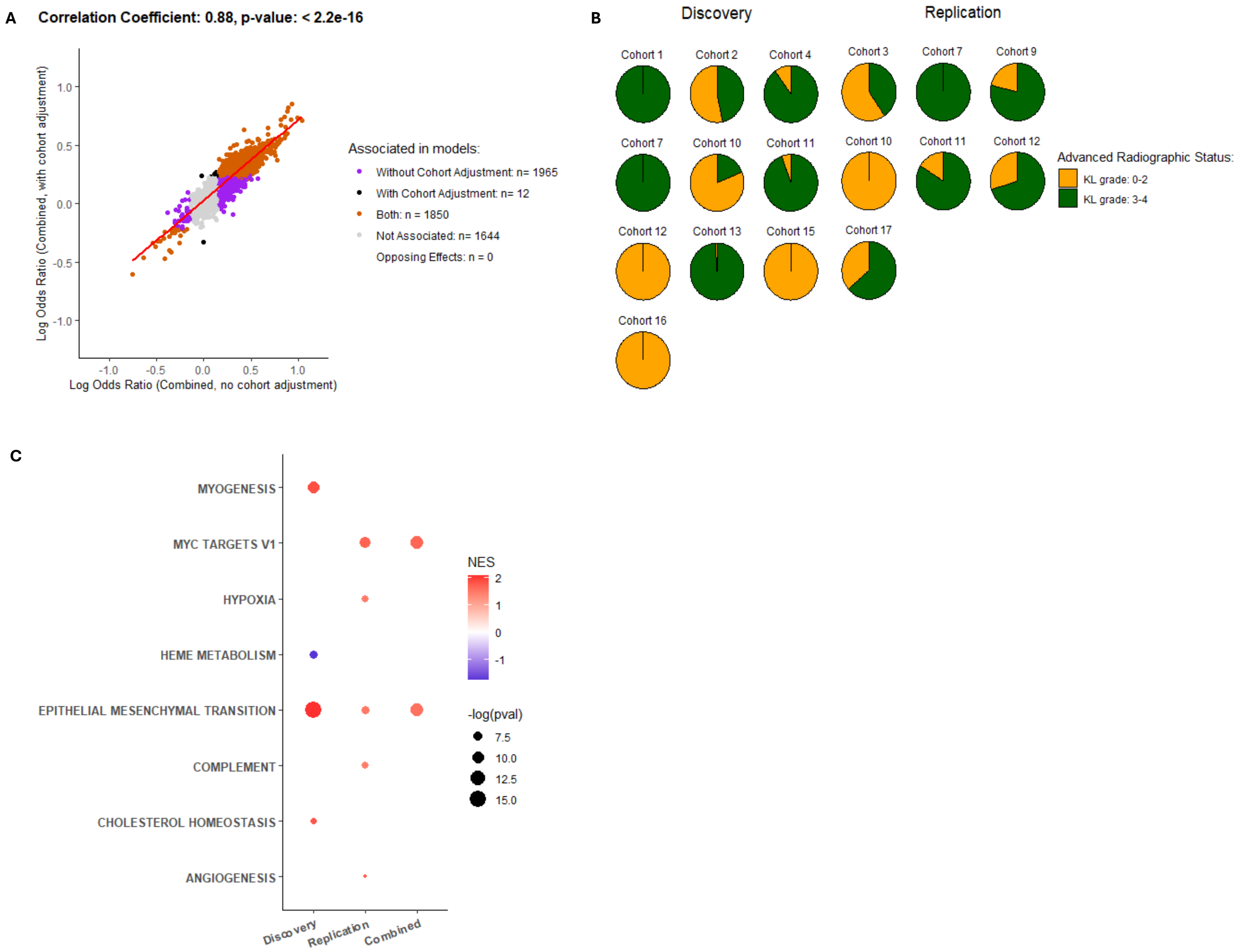

<u>Supplementary Figure 2</u>: ***Association between protein abundance and advanced radiographic knee OA status in the non-IPS regressed data using logistic regression modelling* with additional adjustment for cohort.**

Protein abundance was measured in 1,322 samples (Combined: 1,096 spun, 226 unspun), adjusted for spin-status (using ComBat), and then age, biological sex and cohort (random intercept). **(A)** A scatter plot of log odds ratio (logOR) from logistic regression models of the associations between protein abundance and advanced radiographic disease status using either the Combined dataset without adjustment for cohort or with adjustment for cohort is shown with significantly associated proteins (at padj ≤ 0.05) in different models shown in different colours (see key). Pearson correlation coefficient and p-value (unadjusted) are presented for the correlation between logORs generated in Combined (non-cohort adjusted and cohort adjusted) analyses. **(B)** Pie charts demonstrating the variation in cohort composition of advanced radiographic disease severity across individual cohorts within Discovery and Replication (for baseline OA samples). **(C)** Bubble plot of significantly enriched pathways (padj <0.05) using the Hallmark Gene set for advanced radiographic knee OA status using Discovery, Replication and Combined non-IPS regressed datasets with additional adjustment for cohort (random intercept). Abbreviations: normalised enrichment score (NES); intracellular protein score (IPS); log odds ratio (logOR); Kellgren Lawrence (KL); adjusted p-value (padj). Full list of proteins is available in Source Data file 1.

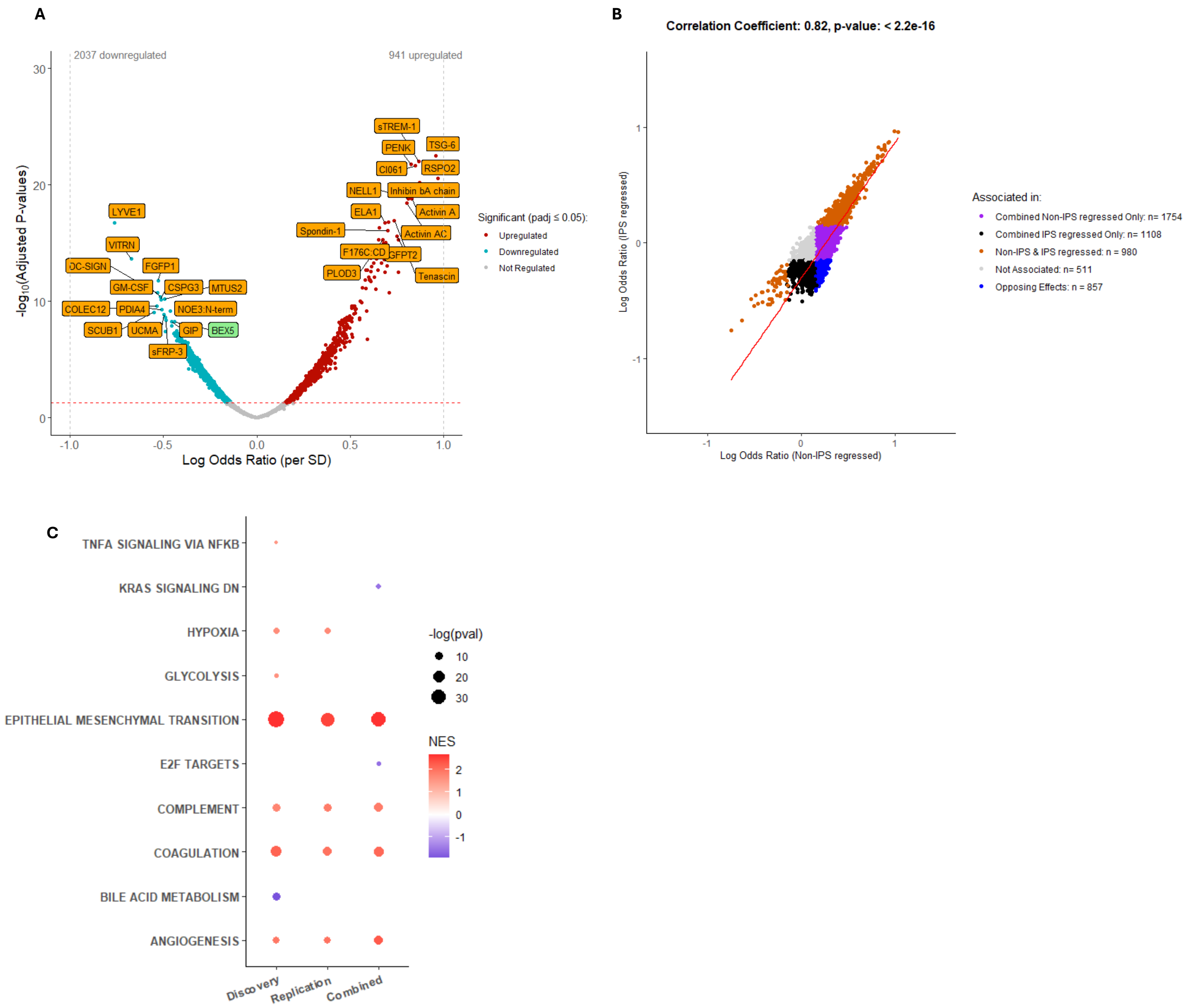

Supplementary Figure 3: ***Association between protein abundance and advanced radiographic knee OA status in the IPS regressed data using logistic regression modelling.***

Protein abundance was measured in 1,322 samples (Combined: 1,096 spun, 226 unspun), adjusted for spin-status (using ComBat), and then age and biological sex. Additionally, these proteomic data underwent IPS regression. **(A)** Volcano plot showing log odds ratios (logOR) between categories per standard deviation change in protein expression for proteins associated with advanced radiographic status (KL grades 3-4) in the Combined dataset, with Benjamini-Hochberg adjusted p-values (padj). Proteins in red are positively associated while those in blue are negatively associated with advanced radiographic status (KL grades: 3-4) at padj ≤ 0.05. Top 30 associated proteins in each direction, by padj, are labelled. In orange are proteins that replicated (significant at padj ≤0.05 and with effects in the same direction, in Discovery & Replication datasets), and remained significant after the Combined dataset was adjusted for cohort (as a random intercept). Proteins that either did not replicate but remained significant after adjustment for cohort, or did replicate but were not significant after cohort adjustment are shown in green. **(B)** Scatter plot of logORs from logistic regression models of the

association between protein abundance and advanced radiographic status in non-IPS regressed and IPS regressed analyses is shown with significantly associated proteins (at padj ≤ 0.05) in different groups shown in different colours (see key). Only proteins common to both IPS and non-IPS regressed analyses were included (N = 5210). Pearson correlation coefficient and p-value (unadjusted) are presented for the correlation between logORs generated in non-IPS and IPS regressed analyses using Combined datasets. **(C)** Bubble plot of significantly enriched pathways (padj <0.05) using the Hallmark Gene set for advanced radiographic disease status using Discovery, Replication and Combined IPS regressed datasets. Abbreviations: normalised enrichment score (NES); intracellular protein score (IPS); log odds ratio (logOR); adjusted p-value (padj). The full list of proteins is available in Source Data file 2.

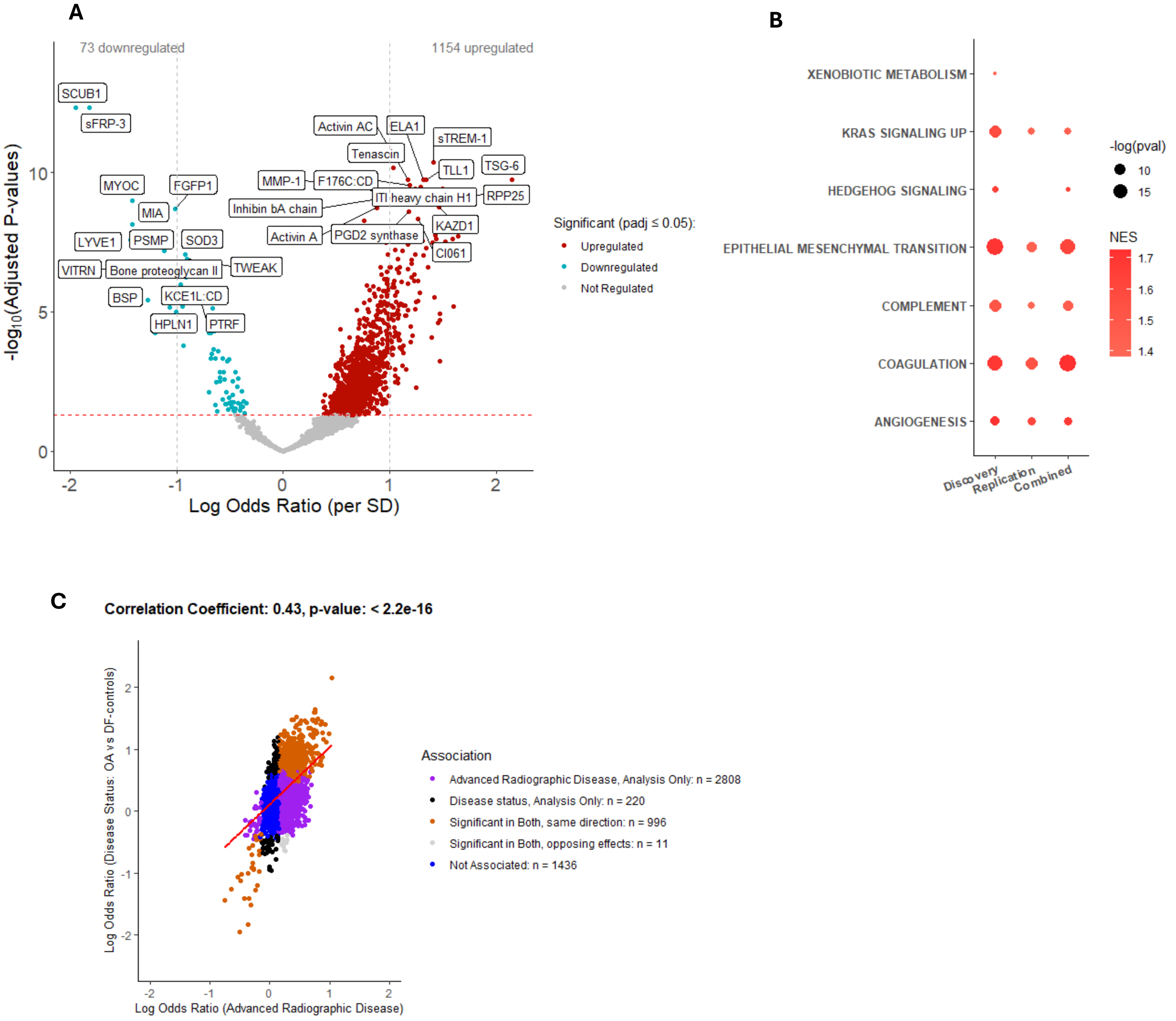

<u>**Supplementary Figure 4**</u>: ***Association between protein abundance and disease status (comparing proteomes of OA and disease-free controls) in Combined, non-IPS regressed data using logistic regression modelling.***

Protein abundance was measured in N = 1361 OA (spun (N = 1,134) and unspun (N = 227)) & N = 36 disease-free control samples (spun (N = 6) and unspun (N = 30)), corrected for spin-status by ComBat and then adjusted for age and biological sex. **(A)** Volcano plot showing log odds ratios (logOR) between categories per standard deviation change in protein expression for proteins associated with disease status in the Combined dataset, with Benjamini-Hochberg adjusted p-values (padj). Proteins in red are positively associated while those in blue are negatively associated with belonging to the OA disease group (compared to DF-controls) at padj ≤ 0.05. Top 30 associated proteins in each direction, by padj,

are labelled. **(B)** Bubble plot of significantly enriched pathways (at padj<0.05) using the Hallmark Gene set for disease status (OA vs DF-controls) for Discovery, Replication and Combined non-IPS, non-cohort adjusted datasets. **(C)** Scatter plot of logORs from logistic regression models of the associations between protein abundance and advanced radiographic disease, and disease status (OA vs DF-controls) using Combined datasets is shown with significantly associated proteins (at padj ≤ 0.05) in different analyses shown in different colours (see key). Pearson correlation coefficient and p-value (unadjusted) are presented for the correlation between logORs generated in advanced radiographic disease and disease status (OA vs DF-controls) analyses. Abbreviations: normalised enrichment score (NES); intracellular protein score (IPS); disease-free (DF); standard deviation (SD); log odds ratio (logOR); adjusted p-value (padj). The full list of proteins is available in Source Data file 3.

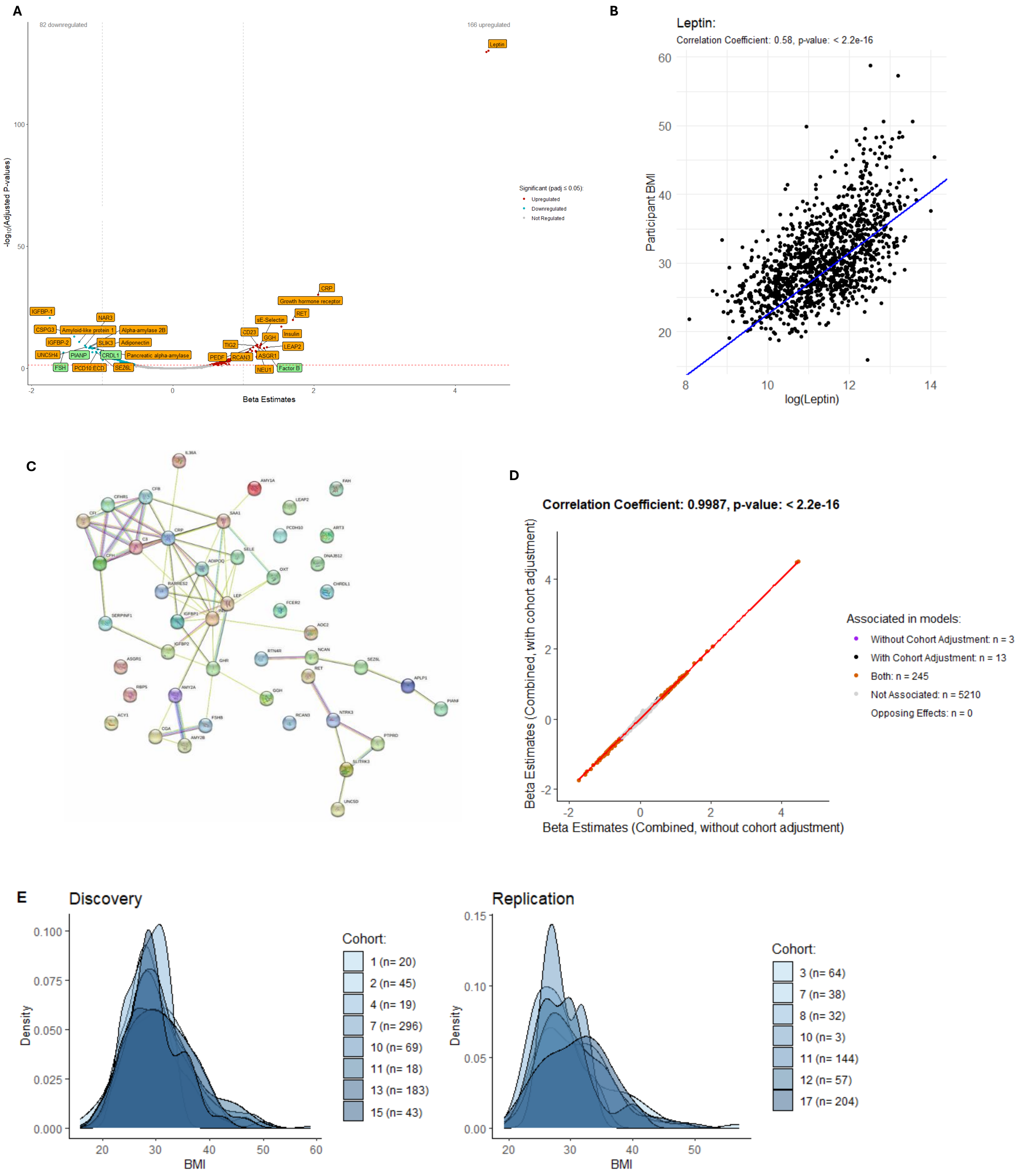

<u>Supplementary Figure 5</u>: *Association between protein abundance and body mass index (BMI) in OA using the non-IPS regressed dataset using linear regression modelling.*

Protein abundance was measured in 1,236 samples where BMI was available (Combined: 1,045 spun, 191 unspun), adjusted for spin-status (using ComBat), and then biological sex and advanced radiographic disease severity. **(A)** Volcano plot showing beta estimates per standard deviation change in protein expression for proteins associated with BMI in the Combined dataset, with Benjamini-Hochberg adjusted p-values (padj). Proteins in red are positively associated while those in blue are negatively associated with increasing BMI at padj ≤ 0.05. Top 30 associated proteins in each direction, by padj, are labelled. In orange are proteins that replicated (significant at padj ≤0.05 and with effects in the same direction in Discovery & Replication datasets), and remained significant after Combined dataset was adjusted for cohort (random intercept). Proteins that either did not replicate but remained significant after adjustment for cohort, or did replicate but were not significant after cohort adjustment are shown in green. **(B)** Scatter plot of participant BMI against log-transformed leptin protein expression. Pearson correlation coefficient and p-value (unadjusted) are presented, with linear regression line plotted. **(C)** Protein-protein interaction network, using STRING, for those proteins associated with BMI (top 50 most significant based on padj). **(D)** Scatter plot of beta estimates from linear regression models of the association between protein abundance and BMI using the Combined dataset without adjustment for cohort or Combined dataset with adjustment for cohort (random intercept) is shown with significantly associated proteins (at padj ≤ 0.05) in different models shown in different colours (see key). Pearson correlation coefficient and p-value (unadjusted) are presented for the correlation between beta estimates generated in Combined analyses with and without adjustment for cohort. **(E)** Distribution of BMI across individual cohorts within Discovery and Replication datasets (only cohorts with more than 2 data points are shown). Abbreviations: body mass index (BMI); intracellular protein score (IPS); adjusted p-value (padj). Full list of proteins is available in Source Data file 4.

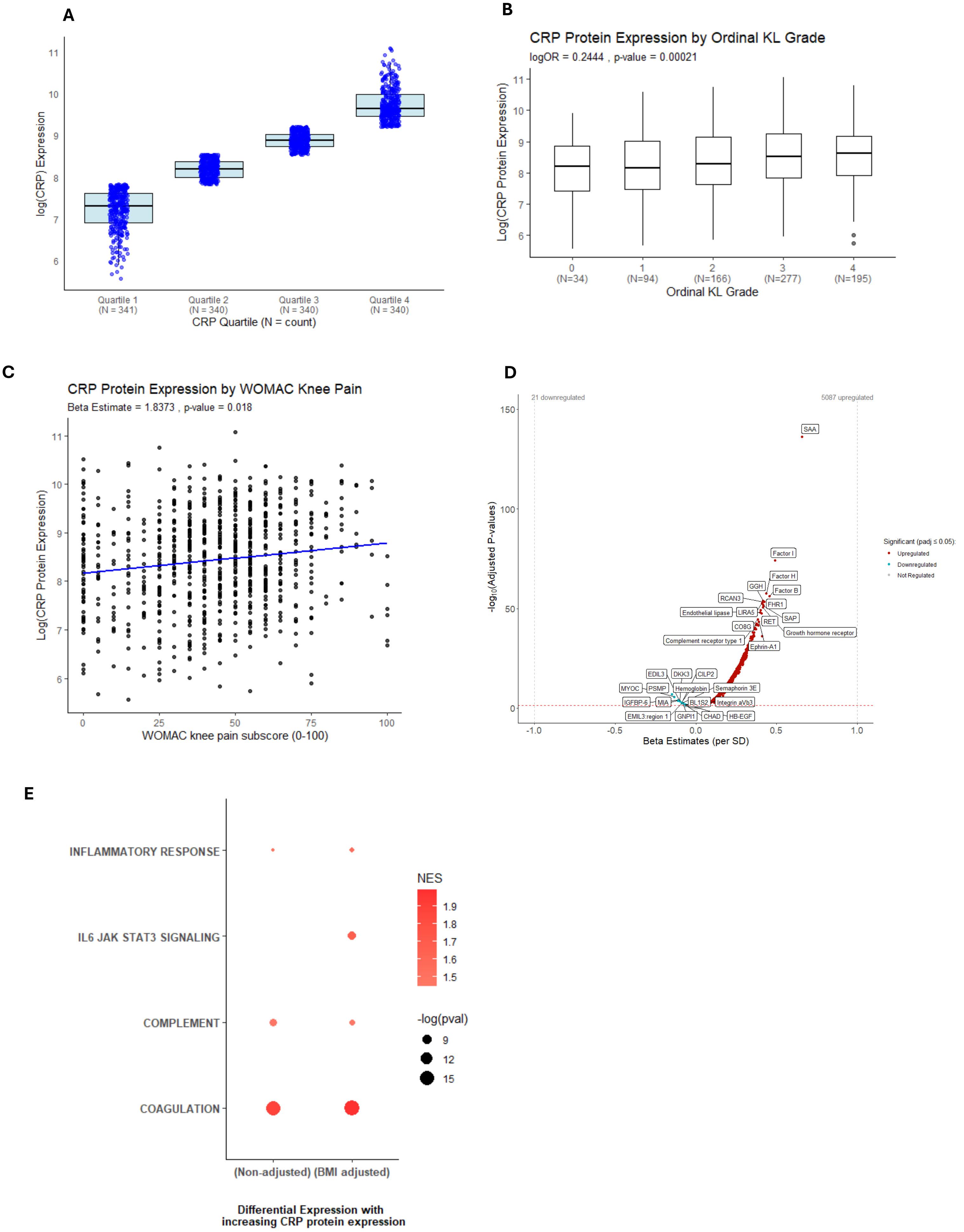
A
log(CRP) Expression
Quartile 1 (N = 341)
Quartile 2 (N = 340)
Quartile 3 (N = 340)
Quartile 4 (N = 340)
CRP Quartile (N = count)
B
CRP Protein Expression by Ordinal KL Grade
logOR = 0.2444 , p-value = 0.00021
Log(CRP Protein Expression)
0 (N=34)
1 (N=94)
2 (N=166)
3 (N=277)
4 (N=195)
Ordinal KL Grade
C
CRP Protein Expression by WOMAC Knee Pain
Beta Estimate = 1.8373 , p-value = 0.018
Log(CRP Protein Expression)
WOMAC knee pain subscore (0-100)
D
21 downregulated
5087 upregulated
SAA
Factor I
Factor H
GGH
Factor B
RCAN3
FHR1
Endothelial lipase
LIRA5
SAP
CO8G
RET
Growth hormone receptor
Complement receptor type 1
Ephrin-A1
EDIL3
DKK3
CILP2
MYOC
PSMP
Hemoglobin
Semaphorin 3E
IGFBP-6
MIA
BL1S2
Integrin aVb3
EMIL3:region 1
GNPI1
CHAD
HB-EGF
$-\log_{10}$(Adjusted P-values)
Beta Estimates (per SD)
Significant (padj ≤ 0.05):
Upregulated
Downregulated
Not Regulated
E
INFLAMMATORY RESPONSE
IL6 JAK STAT3 SIGNALING
COMPLEMENT
COAGULATION
NES
-log(pval)
(Non-adjusted) (BMI adjusted)
Differential Expression with increasing CRP protein expression

__Supplementary Figure 6__: ***Association between protein abundance and C-reactive protein (CRP) in OA individuals in non-IPS regressed, Combined data using linear regression modelling.***
Protein abundance for N = 5470 proteins was measured in 1,361 OA samples (Combined dataset, spun (N = 1,134) and unspun (N = 227)), corrected for spin-status (by ComBat). **(A)** CRP protein expression (transformed by natural logarithms) by quartiles. **(B)** CRP protein expression (transformed by natural logarithms) by ordinal KL grade (N = 766 samples). Statistically significant associations with ordinal KL grade were tested by proportional odds ordinal regression (log odds ratio (logOR) and unadjusted p-values are presented for the model adjusted for age and biological sex). Number of samples for each level of radiographic disease (KL grading) are shown. Boxplots show median, interquartile range, and whiskers representing the most extreme values within 1.5 times the interquartile range, with outliers plotted individually. **(C)** Scatter plot of WOMAC knee pain subscores against CRP protein abundance (transformed by natural logarithms) (N = 805 samples). Beta estimates and p-values (unadjusted) are presented for a linear regression model adjusted for age, biological sex and advanced radiographic disease status. **(D)** Volcano plot showing beta estimates per standard deviation change in protein expression for proteins associated with natural log transformed CRP protein expression in the Combined dataset, with Benjamini-Hochberg adjusted p-values (padj), adjusted for age, biological sex and advanced radiographic disease status. Proteins in red are positively associated, those in blue negatively associated, with increasing log(CRP) at padj ≤ 0.05. Top 30 associated proteins in each direction, by padj, are labelled. **(E)** Bubble plot of significantly enriched pathways (padj <0.05) using the Hallmark Gene set for proteins associated with CRP using Combined dataset with and without additional adjustment for BMI ('non-adjusted' model: adjusted for age, biological sex and advanced radiographic disease; 'adjusted' model: adjusted for age, biological sex, advanced radiographic disease and BMI). Abbreviations: intracellular protein score (IPS); log odds ratio (logOR); Western Ontario and McMaster Universities Osteoarthritis Index (WOMAC); C-reactive protein (CRP); body mass index (BMI); standard deviation (SD); adjusted p-value (padj). Full list of proteins is available in Source Data file 6.

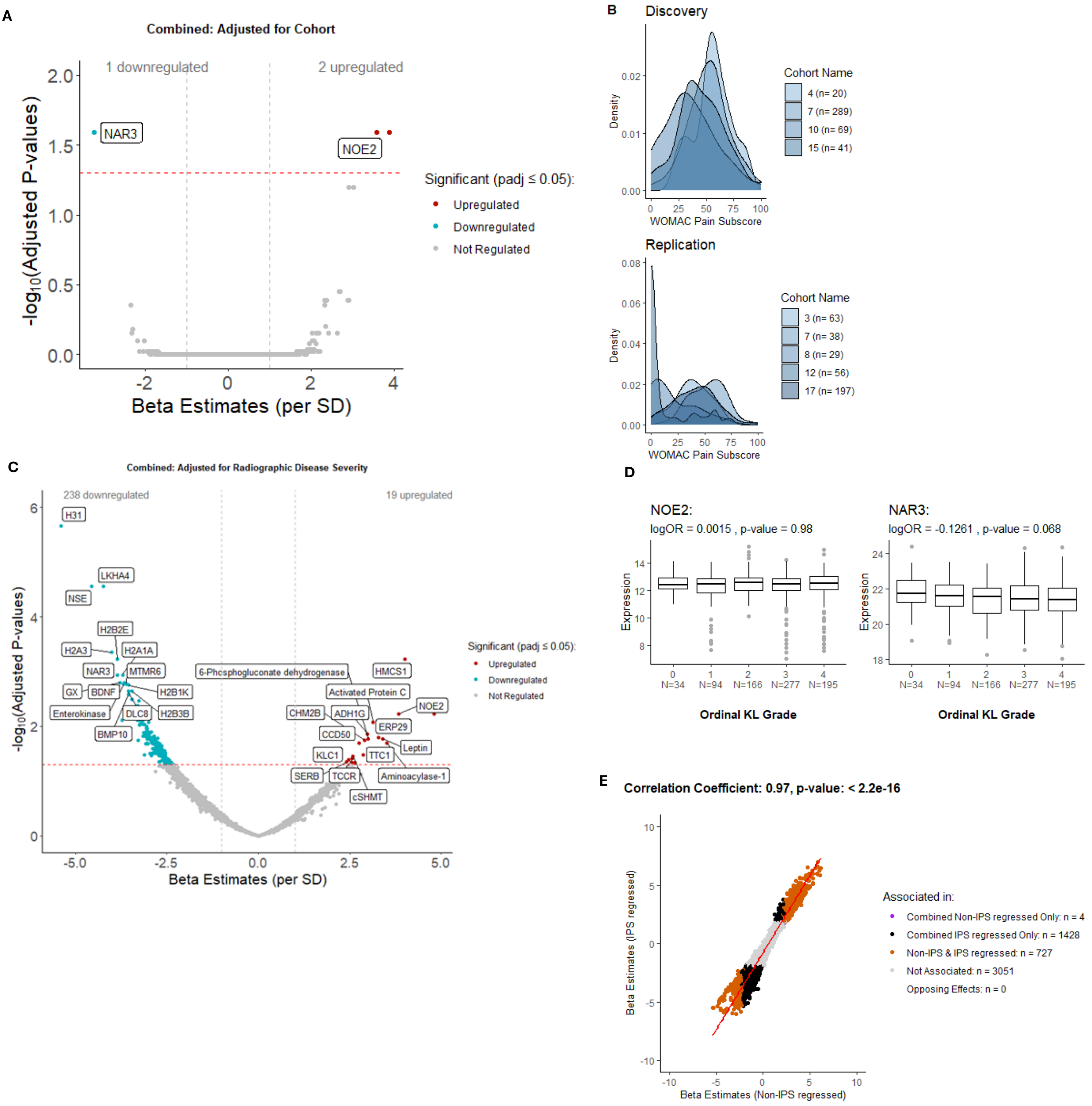

<u>Supplementary Figure 7</u>: *Association between protein abundance and WOMAC knee pain subscores in the non-IPS regressed data using linear regression modelling after adjustment for cohort or advanced radiographic disease status.*

Protein abundance was measured in 805 OA samples where WOMAC knee pain subscore was available in the Combined dataset (spun (N = 748) and unspun (N = 57)). **(A)** Volcano plot showing beta estimates per standard deviation against adjusted p-values (Benjamini-Hochberg adjusted, padj) for

proteins associated with an increase or decrease in WOMAC knee pain subscore in the Combined dataset adjusted for age, biological sex and cohort (random intercept). Two proteins, labelled in white, remained significantly associated after adjusting for cohort (NOE2 was positively associated and NAR3 negatively associated), at padj ≤ 0.05. **(B)** Variable distribution of WOMAC knee pain subscores is shown for individual cohorts across Discovery and Replication (only cohorts with more than 2 data points are shown). **(C)** Volcano plot showing beta estimates per standard deviation change against adjusted p-values (Benjamini-Hochberg adjusted, padj) for proteins associated with increasing or decreasing WOMAC pain subscore in the Combined dataset, adjusted for age, biological sex and advanced radiographic disease. Top 30 associated proteins in each direction, by padj, are labelled. **(D)** Boxplot of NOE2 or NAR3 protein abundance (transformed by natural logarithms) by ordinal KL grade. Associations with ordinal KL grade were tested by proportional odds ordinal regression analysis (logOR) and unadjusted p-values are presented for each protein for models adjusted for age and biological sex. Boxplots show median, interquartile range, and whiskers representing the most extreme values within 1.5 times the interquartile range, with outliers plotted individually. **(E)** Scatter plot of beta estimates from linear regression models of the associations between protein abundance and WOMAC knee pain using the Combined dataset with and without adjustment for IPS is shown with significantly associated proteins in different datasets shown in different colours (see key). Pearson correlation coefficient and p-value (unadjusted) are presented. Only proteins common to both IPS and non-IPS regressed analyses were included (N = 5210). Abbreviations: osteoarthritis (OA), standard deviation (SD), intracellular protein score (IPS), Western Ontario and McMaster Universities Osteoarthritis Index (WOMAC, 0 = no pain, 100 = worst possible pain); adjusted p-value (padj). The full list of proteins is available in Source Data files 8 & 9.
